# Real-time precise point positioning-based zenith tropospheric delay for precipitation forecasting

**DOI:** 10.1038/s41598-018-26299-3

**Published:** 2018-05-21

**Authors:** Qingzhi Zhao, Yibin Yao, Wanqiang Yao, Zufeng Li

**Affiliations:** 1 College of Geomatics, Xi’an University of Science and Technology, Xi’an, 710054 China; 2 School of Geodesy and Geomatics, Wuhan University, Wuhan, 430072 China; 3 Powerchina Northwest Engineering Corporation Limited, Xi’an, 710065 China

## Abstract

GPS-based Zenith Tropospheric Delay (ZTD) estimation should be easily obtained in a cost-effective way, however, the most previous studies focus on post-processed ZTD estimates using satellite orbit and clock products with at least 3–9 hours latency provided by International GNSS Service (IGS), which limits the GNSS meteorological application for nowcasting. With the development of IGS’s real-time pilot project (RTPP), this limitation was removed by April, 2013 as real-time satellite orbit and clock products can be obtained on-line. In this paper, on the one hand, the GPS-derived ZTD estimation was evaluated using the IGS final and real-time satellite products based on independently developed PPP software. On the other hand, the analysis of the time series of GPS-derived ZTD by least-square fitting of a broken line tendency for a full year of observations, and a forecasting method for precipitation is proposed based on the ZTD slope in the ascending period. The agreement between ZTD slope and the ground rainfall records suggested that the proposed method is useful for the assisted forecasting, especially for short-term alarms.

## Introduction

Global Navigation Satellite System (GNSS) meteorology was proposed in 1990s, and has proved to be a powerful tool in atmospheric water vapour research from micro-meteorology to global climate studies^1–4^. According to the influence of neutral atmospheric delay effect when the satellite signals propagate through the troposphere, the precise atmospheric delay, which is also called zenith total delay (ZTD), can be calculated based on phase observations^5^. The zenith hydrostatic delay (ZHD) can be calculated with high accuracy using the surface pressure^6^. Therefore, the precipitable water vapour (PWV) can be converted from zenith wet delay (ZWD) which is extracted from ZTD by removing ZHD^1^. PWV is the average water vapour content from many signals with different elevation angles and azimuths projected to the vertical direction using the mapping function^7^.

Currently, on the one hand, the tropospheric parameters are estimated based on double differenced observations^8–11^ which has an unfavourable impact. Due to the short distance between the ground-based stations in a region, only relative tropospheric parameters can be obtained without introducing stations with a baseline length of greater than 500 km^3^: however, considering the assistance derived from the use of some stations with a long baseline will enhance the complexity of the observation equation modelling as well as the resolving time issues therein. On the other hand, the precise point pointing technique based on un-differenced observation can be used to obtain absolute tropospheric parameters with only one station and allows the monitoring of typhoons and storms based on the PPP-derived PWV^12–15^.

Although the absolute ZTD/PWV value can be derived from the PPP technique, how to obtain those tropospheric parameters steadily without time latency remains a challenge. A series of studies have been performed to obtain the near real-time ZTD/PWV parameters using the International Global Navigation Satellite System (GNSS) Service (IGS) ultra-rapid (IGU) orbit and clock products with a latency of 3 to 9 h^12,16–19^ however, such time latency makes the application of GNSS meteorology difficult in the real-time monitoring and forecasting of extreme weather events. With the developments made in the IGS real-time pilot project (RTPP)^20^, precise clock and orbit products without time latency can be downloaded on-line^21^, which promotes the rapid development of real-time PPP techniques and makes it easy to obtaining real-time ZTD/PWV products^22–25^. The real-time service (RTS) of IGS officially has provided GPS real-time satellite orbit and clock corrections on 1 April, 2013 at a global scale^21^.

Many research aspects of GNSS meteorology focus on the relationship between PWV and precipitation^2,26–31^ which need the supporting of meteorological data. On the one hand, this requires ground-based stations equipped with meteorological sensors, which increases the operating costs. On the other hand, the conversion error from ZTD to PWV will also be introduced. Unfortunately, most ground-based stations are not equipped with meteorological sensors: this makes many previous studies difficult to apply in reality. Therefore, the focus here was on the study of the relationship between real-time GPS-derived ZTD and precipitation, which exploits the potential of GPS-derived ZTD values using real-time orbit and clock products for precipitation monitoring and forecasting.

In the first part of this paper, the accuracy of post-processed ZTD derived from the independent developed PPP software was compared with the ZTD derived from GAMIT, Bernese PPP model and VLBI, the real-time satellite orbit and clock products provided by RTS were also evaluated with respect to the IGS final orbit and clock products and the real-time GPS-derived ZTD was compared with the post-processed GPS-derived ZTD. In the second part, the relationship between hourly ZTD and precipitation was analysed in different weather conditions and a simple precipitation forecasting method is proposed based on one full year of ZTD time series data. Finally, conclusions and discussions are provided.

## Tropospheric delay parameter: ZTD

The propagated delay of satellite signals in the low atmosphere layer is mainly affected by atmospheric delay effect, which can be divided into the hydrostatic part and the wet part^32,33^.

1 $${\rm{STD}}={\rm{SHD}}+{\rm{SWD}}$$

where ${\rm{STD}}$ is the slant total delay, ${\rm{SHD}}$ is the slant hydrostatic delay, and ${\rm{SWD}}$ is the slant wet delay. The STD can be expressed by projecting ZTD in the zenith direction of a station along the signal direction.

2 $${\rm{STD}}=f(ele,azi)\cdot {\rm{ZTD}}$$

where *f* refers to the mapping function while *ele* and *azi* represent the elevation angle and azimuth angle, respectively. Many practical mapping functions, such as NMF, GMF and VMF1, can be used to obtain an accurate STD when neglecting the influence of satellite ray-bending^34–36^. ZTD is the average delay value of many signals derived from the same receiver and can be expressed by:

3 $${\rm{ZTD}}={\rm{ZHD}}+{\rm{ZWD}}$$

where ZHD is the zenith hydrostatic delay, about 90% of ZTD, mainly affected by the latitude of station and surface pressure^6^. ZWD is the zenith wet delay, about 2% to 20% of ZTD, which affects the propagation of satellite signals by the movements of the poles of water vapour molecules and is largely related to the water vapour concentration. Before a few hours of precipitation, ZTD is also disturbed by the zenith delay of hydrometeors (ZHMD) which accounts for up to 3% of ZTD. ZHMD is caused by liquid water and icy hydrometeors, the latter being about the one-tenth of the former. During the rainy days, ZHMD reaches its maximum with a value of 0.07 m and is mainly caused by the convection activities of precipitation weather^5,37^.

## The retrieval of ZTD and accuracy-testing

As mentioned before, the tropospheric parameters can be estimated by un-differenced or double-differenced means, and GNSS observation processing software has been developed based thereon, such as: GAMIT/GOBK, Bernese, GIPSY, EPOS, TriP, etc. In our study, an independently developed PPP software was used to process the GPS observations. To validate the accuracy and reliability of PPP software developed by ourselves, the estimated ZTD was compared with that from GAMIT/GLOBK (v10.5) software, the PPP module of Bernese software, as well as that from very long baseline interferometry (VLBI).

### Data and processing strategy

**Figure 1:**
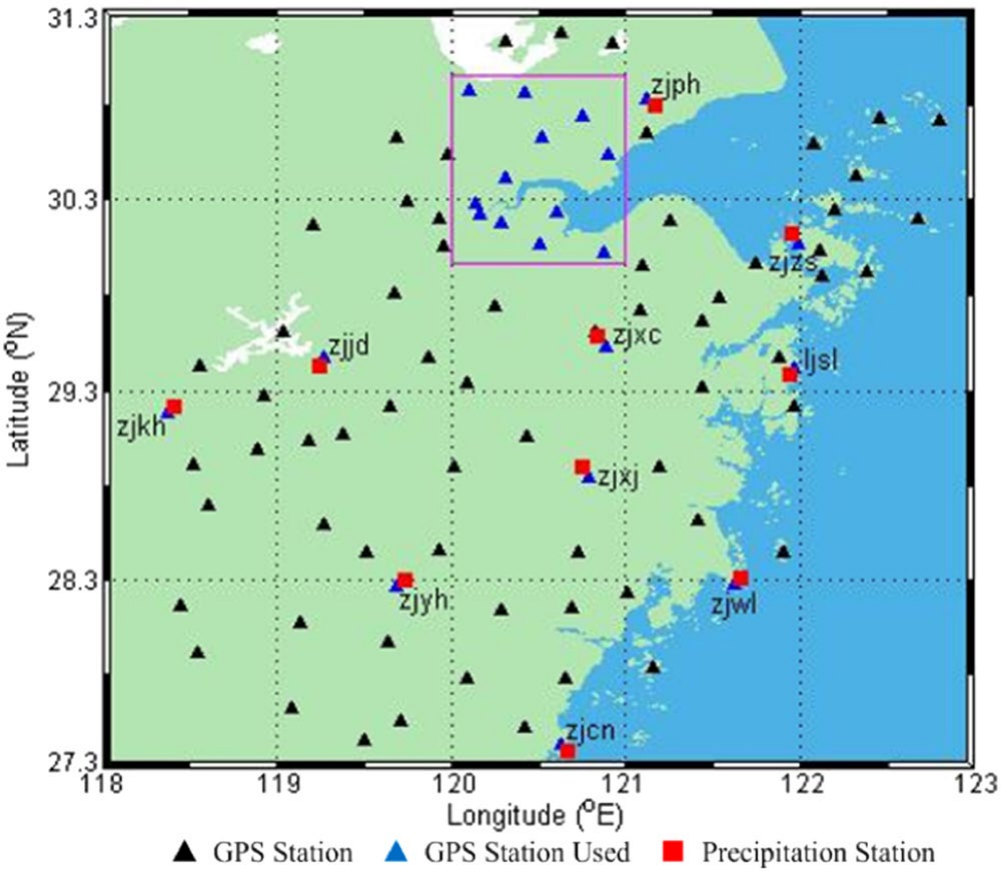
Geographical location of GNSS, and precipitation, stations in the CORS network of Zhejiang Province. [the figure is plotted by MATLAB 2016a (https://cn.mathworks.com/products/matlab.html)].

A full year of GPS data of ten GPS stations (as shown by the blue triangles outside the magenta rectangle in Fig. 1) from the Continuously Operating Reference Stations (CORS) network of Zhejiang Province, China from 1 September, 2014 to 31 August, 2015 was selected. The precipitation information of nearby rainfall stations at ten ground-based GNSS stations with a temporal resolution of 1 h was also accumulated so as to analyse the relationship between ZTD time series data and precipitation. The geographic distribution of receivers and rainfall stations is shown in Fig. 1. In addition, two-day’s data from twelve GPS stations (as shown by the blue triangles inside the magenta rectangle in Fig. 1) and nearby rainfall stations for the period 17–18, June, 2015 were also selected to reflect the 2-d image of ZTD during the cold front prevailing in the north of Zhejiang Province.

The GPS observations were processed with a sampling interval of 30 seconds while the elevation angle mask was selected as 10°. Unfortunately, some of the selected GPS stations (as presented by the blue triangles inside the magenta rectangle in Fig. 1) were not equipped with meteorological sensors, and the accurate meteorological parameters for those stations cannot be obtained. Therefore, the ZHD is calculated for those stations based on the global pressure and temperature 2 (GPT2) model using the formula proposed by Saastamoinen (1972):

4 $${\rm{ZHD}}=\frac{0.002277\cdot {P}_{s}}{1-0.00266\cdot \,\cos (2\phi )-0.00028\cdot H}$$

where ${P}_{s}$ is the surface pressure, and $\phi$ and *H* are the latitude and height of the station. ZWD is mainly determined by water vapour content which cannot be calculated by the empirical model, so ZWD is estimated in a random walk pattern together with other unknown parameters. The antenna phase centre offset and variation are corrected using the absolute phase centre correction model^38^. The global mapping function is adapted for the tropospheric mapping function. In this paper, the real time stream of CLK93 was used to obtain the orbit and clock information. The unknown parameters (receiver coordinates, troposphere zenith wet delay, receiver clock error, etc.) are estimated using the extended Kalman Filter.

After the ZTD and ZHD are obtained, the PWV can be calculated using the following formula:

5 $${\rm{PWV}}={\rm{\Pi }}\cdot {\rm{ZWD}}$$

where Π refers to the conversion factor while ZWD can be obtained by extracting ZHD from ZTD. Π can be calculated based on the formula

6 $${\rm{\Pi }}=\frac{{10}^{6}}{({k}_{2}^{^{\prime} }+{k}_{3}/{T}_{m})\cdot {R}_{v}\cdot \rho }\cdot $$

where ${k}_{2}^{^{\prime} }=16.48\,K\cdot hP{a}^{-1}$ and *k*_3_ = (3.776 ± 0.014) × 10^5^ *K*^2^ · *hPa*^−1^ are constants, ${R}_{\omega }=461\,(J\cdot k{g}^{-1}\cdot {K}^{-1})$ represents the ideal gas constant for water vapour, *ρ* is the density of the water vapour density, *T*_*m*_ is the mean temperature of the atmospheric colum.

### Evaluation of post-processed PPP-derived ZTD

Previous studies have proved that the accuracy of absolute ZTD derived from GAMIT/GLOBK software based on double-differenced observations was better than ±1 cm^39^ while the internal accuracy of ZTD derived from PPP module of the Bernese software based on un-differenced observations was better than ±1.3 mm^40^ with the mean bias of 7 mm^41^. Therefore, the ZTD parameter estimated from two softwares mentioned above is considered as a reference to evaluate the developed PPP software.

**Figure 2:**
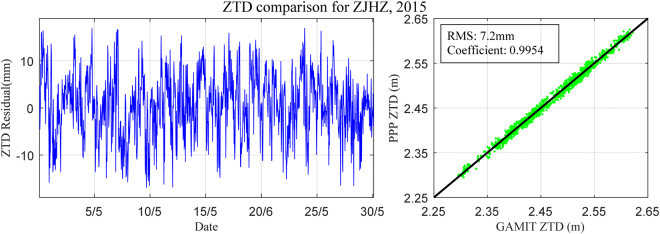
ZTD residuals time series derived from GAMIT and PPP: 1 May to 31 May, 2015. [the figure is plotted by MATLAB 2016a (https://cn.mathworks.com/products/matlab.html)].

**Figure 3:**
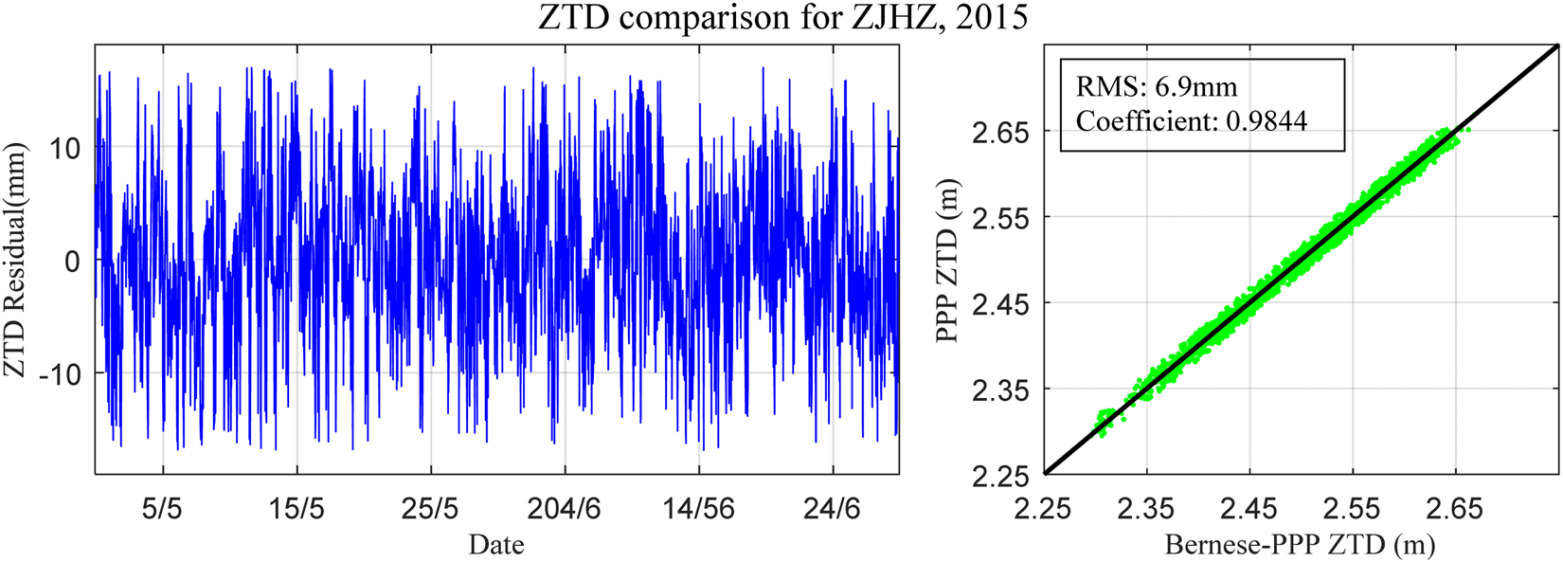
ZTD residual time series derived from Bernese PPP and PPP: 1 May to 30 June, 2015. [the figure is plotted by MATLAB 2016a (https://cn.mathworks.com/products/matlab.html)].

The ZTD of ZJHZ station derived from GAMIT/GLOBK software was selected for the period 1–31 May, 2015 to compare with that estimated using the developed PPP software. One point should be noted that, for the ZTD resolution over a region (as shown by the magenta rectangle in Fig. 1) using GAMIT, three IGS stations (BJFS, LHAZ and SHAO) were also used to reduce the influence of the strong correlation of tropospheric parameters^3^. In addition, the GPS observations at station ZJHZ for the period 1 May to 30 June, 2015 were also processed using the PPP module of the Bernese software and compared with that derived from the developed PPP software. Figures 2 and 3 show the ZTD residual time series derived from the GAMIT and Bernese software for two periods with time intervals of 30 seconds: the ZTD time series derived from different methods were in mutual agreement. Comparing the ZTD derived from GAMIT and Bernese software, the coefficient and RMS error of the developed PPP software were 0.9954/7.2 mm and 0.9844/6.9 mm, respectively. The numerical results show that the developed PPP software is good enough to obtain ZTD parameters with high accuracy and reliability.

### Comparison between post-processed GPS-derived ZTD and VLBI-derived ZTD

**Figure 4:**
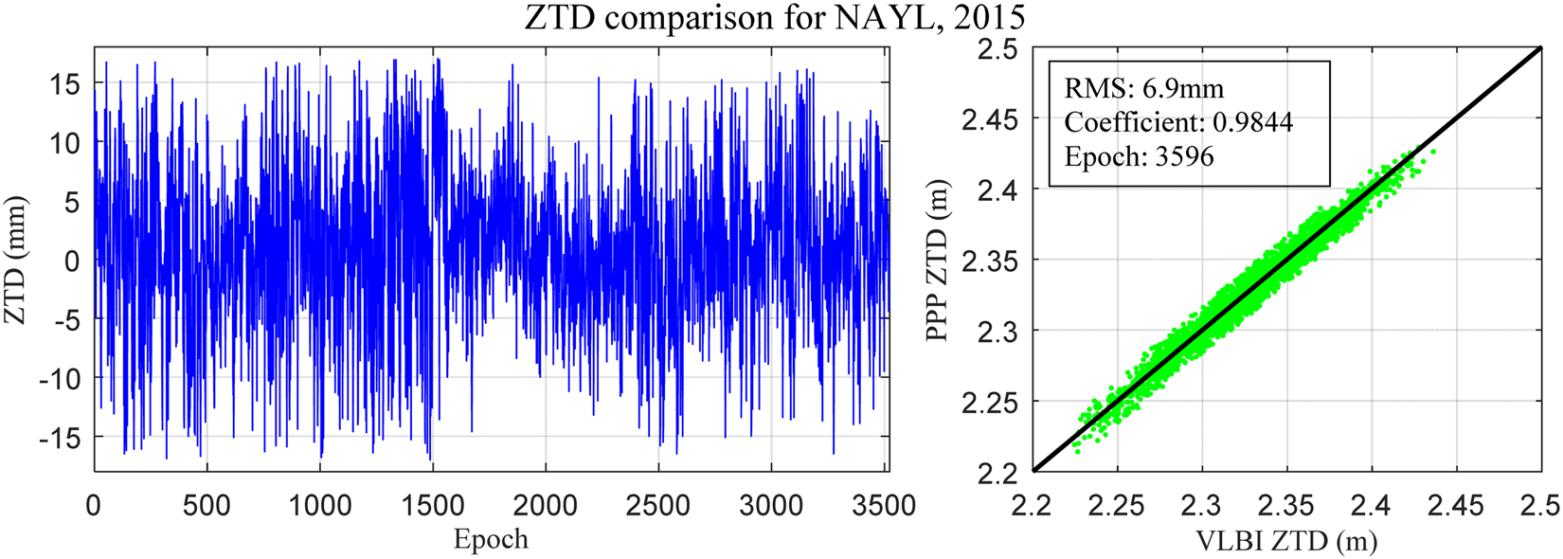
Comparison of ZTD residuals derived from GPS and VLBI at collocated station NAYL:2013 and 2014. [the figure is plotted by MATLAB 2016a (https://cn.mathworks.com/products/matlab.html)].

Researchers have been shown that the ZTD differences between VLBI and GPS were smaller than 1 cm^42,43^ and the VLBI technique was one of the most accurate method of obtaining the ZTD^7^. Therefore, the post-processed GPS-derived ZTD data were compared with another collocated independent technique: Very Long Baseline Interferometry (VLBI). For this comparison, the GPS data of NAYL station and the collocated VLBI station for two years from 2013 to 2014 were selected. Figure 4 shows ZTD residual time series derived from VLBI and GPS based on the developed PPP software at located station NAYL (Norway). Statistical result shows that ZTD derived from the post-processed GPS stations and the collocated VLBI observations are consistent with a coefficient and RMS of 0.9844 and 6.9 mm, respectively. The above result is also clear evidence that the developed PPP software is able to be used to provide precise ZTD parameters.

### Evaluation of the real-time satellite orbit and clock products

The real-time combined orbit and clock products provided by IGS TRS can be obtained on-line, and has combined both single-epoch and Kalman filter approaches. The essential difference of real-time PPP is what kind of orbit and clock information used for processing the observations with respect to the post-processed PPP technique. The nominal accuracies of IGS final orbit and clock products are 2.5 cm and 0.075 ns, separately^44^. To assess the accuracy of the IGS real-time orbit and clock products, the consecutive corrections of orbit and clock products for twenty-five days were selected from 25 June to 19 July, 2015. The coordinate difference between the real-time orbit and the final orbit for each satellite was calculated and then the RMS error of orbit accuracy was obtained. Due to the inconsistency of the clock system between the real-time, and final, clock products, a systemic bias was found. Here, one reference satellite with pseudo-random noise (PRN) #1 was selected for each of the clock system to combine a single difference so as to exclude such bias and the RMS error of single-differenced error between the real-time, and final clock products was obtained.

**Figure 5:**
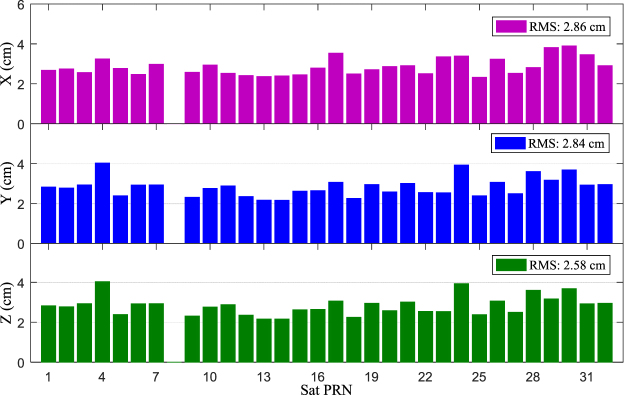
Statistical coordinate accuracy between IGS real-time and final orbit product from 25 June to 19 July, 2015. [the figure is plotted by MATLAB 2016a (https://cn.mathworks.com/products/matlab.html)].

**Figure 6:**
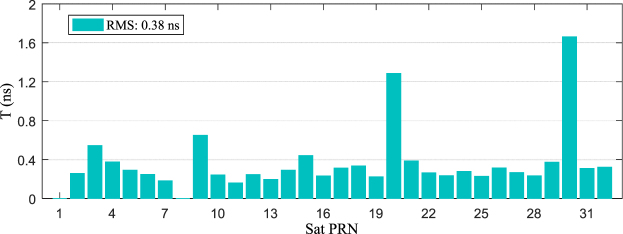
Statistical clock accuracy between IGS real-time and final orbit product from 25 June to 19 July, 2015. [the figure is plotted by MATLAB 2016a (https://cn.mathworks.com/products/matlab.html)].

Figures 5 and 6 show the comparison of the result obtained from the IGS real-time and final products (orbit and clock). On the one hand, the statistical accuracy of the satellite position in the X-, Y-, and Z-directions were 2.86, 2.84, and 2.58 cm, respectively, and the three-dimensional accuracy of the IGS real-time satellite was 4.79 cm. On the other hand, the statistical real-time clock errors with accuracy of 0.38 ns compared to the IGS final clock product, which is superior to the IGS-provided ultra-rapid clock product with the nominal value of approximately 3 ns (http://igscb.jpl.nasa.gov/components/prods.html).

### Comparison of GPS-derived ZTD time series using IGS real-time and final orbit and clock products

Here, the real-time GPS-derived ZTD time series were analysed compared to the ZTD derived from the post-processed GPS-derived ZTD based on the independently developed PPP software. Six-months of GPS observations from stations ZJJD and ZJZS (parts of the Crustal Movement Observation Network of China) were collected with a sampling rate of 30 seconds from 1 February to 31 July, 2015 and processed. Hourly ZTD time series derived from PPP technique using real-time and final orbit and clock correction were obtained and compared.

**Figure 7:**
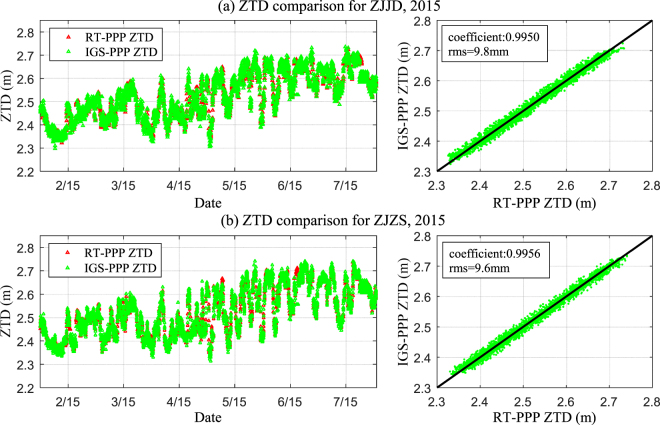
Comparison of GPS PPP-derived ZTD time series using real-time and final orbit and clock products: 1 February to 31 July, 2015. [the figure is plotted by MATLAB 2016a (https://cn.mathworks.com/products/matlab.html)].

Figure 7 shows the time series plot of ZTD derived from two methods mentioned above. Among all those experimental data, some data are apparently faulty, thus a principle is needed with which to exclude incorrect ZTD pairs. In our study, the mean and RMS error were calculated for the differences between the two PWV time series, and then, the final data was selected using those differences less than three-times the RMS error. It can be seen from Fig. 7 that, compared to the GPS-derived ZTD based on IGS final products, the correlation coefficient and RMS error of real-time GPS-derived ZTD at stations ZJJD and ZJZS were 0.9954/7.2 mm and 0.9959/7.8 mm, respectively. This showed that the real-time GPS-derived ZTD time series had good consistency with the post-processed result. Therefore, it is reasonable to replace post-processed ZTD by real-time ZTD for precipitation analysis and further consider real-time ZTD for precipitation monitoring and forecasting.

## Relationship between ZTD and precipitation

### Feasibility of replacing PWV by ZTD for precipitation forecasting

**Figure 8:**
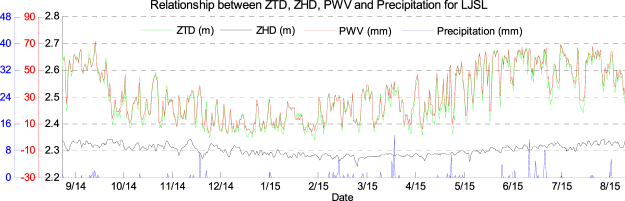
Time series of hourly ZTD, ZHD, PWV, and accumulated rain for LJSL: 1 September, 2014 to 31 August, 2015. [the figure is plotted by MATLAB 2016a (https://cn.mathworks.com/products/matlab.html)].

The water vapour content in the lower atmosphere will increase before precipitation, which increases the atmospheric delay effect on the satellite signal as expressed by an increasing ZTD/PWV ratio. Currently, most studies focus on the analysis of the relationship between PWV and precipitation^26–31^ however, such studies need the support of accurate measured meteorological data to guarantee the accuracy of conversion from ZTD to PWV. Therefore, an idea was proposed in this paper that, if the variation trend in the PWV time series is the same as that of the ZTD time series, then the PWV can be replaced by ZTD for analysis and forecasting of precipitation. The merits of such idea are that: (1) the estimation of ZTD does not requires the participation of meteorological parameters, which avoids a loss of accuracy due to measurement error in any weather equipment and the conversion error from ZTD to PWV; (2) ground-based receivers do not need to be equipped with meteorological sensors which decreases the equipment, and running, costs. Therefore, whether the variation trends of ZTD and PWV are the same or not is first explored in this section with a full year of GPS data from station LJSL from 1 September, 2014 to 31 August, 2015. This station was selected for (1) its proximity to the ocean and the variation in the water vapour content in the atmosphere is large, therefore, the results is more convincing; (2) in addition, the observed meteorological parameters (temperature and pressure) can be obtained for LJSL station. The GPS data of station LJSL was processed by the newly developed PPP software using the IGS final orbit and clock products to obtain the estimated ZTD, ZHD, and PWV on an hourly basis. In the meantime, the hourly precipitation data from nearby (for one full year) was also selected for the later analysis of the relationship between precipitation and ZTD, ZHD, and PWV. Figure 8 shows the time series of ZTD, ZHD, PWV, and precipitation at station LJSL at 1 hour-intervals for the period 1 September, 2014 to 31 August, 2015. Figure 8 shows that the trends in ZTD and PWV were similar and their coefficient was 0.9666, while the trends of ZHD appeared irrelevant with that from ZTD/PWV time series. The maximum variations of ZHD and ZTD were 0.1 and 0.37 m, respectively. It may be reasonable to consider that most of the variation in ZTD was caused by variations in the wet delay or water vapour content. Therefore, it is acceptable to replace PWV by ZTD and use it as an indicator to reflect the variation of precipitation.

### Relationship between ZTD and precipitation

To analyse the relationship between ZTD and precipitation during rainfall, one full year of hourly ZTD and hourly accumulated precipitation near station ZJZS (less than 2 km away) from 1 September, 2014 to 31 August, 2015 were selected. In addition, ten-minute ZTD was also obtained for calculating the ZTD increment. In this section, two cases of rainfall were selected at station ZJZS from 6 to 11 April, and 9 June to 11 June, 2015, respectively.

**Figure 9:**
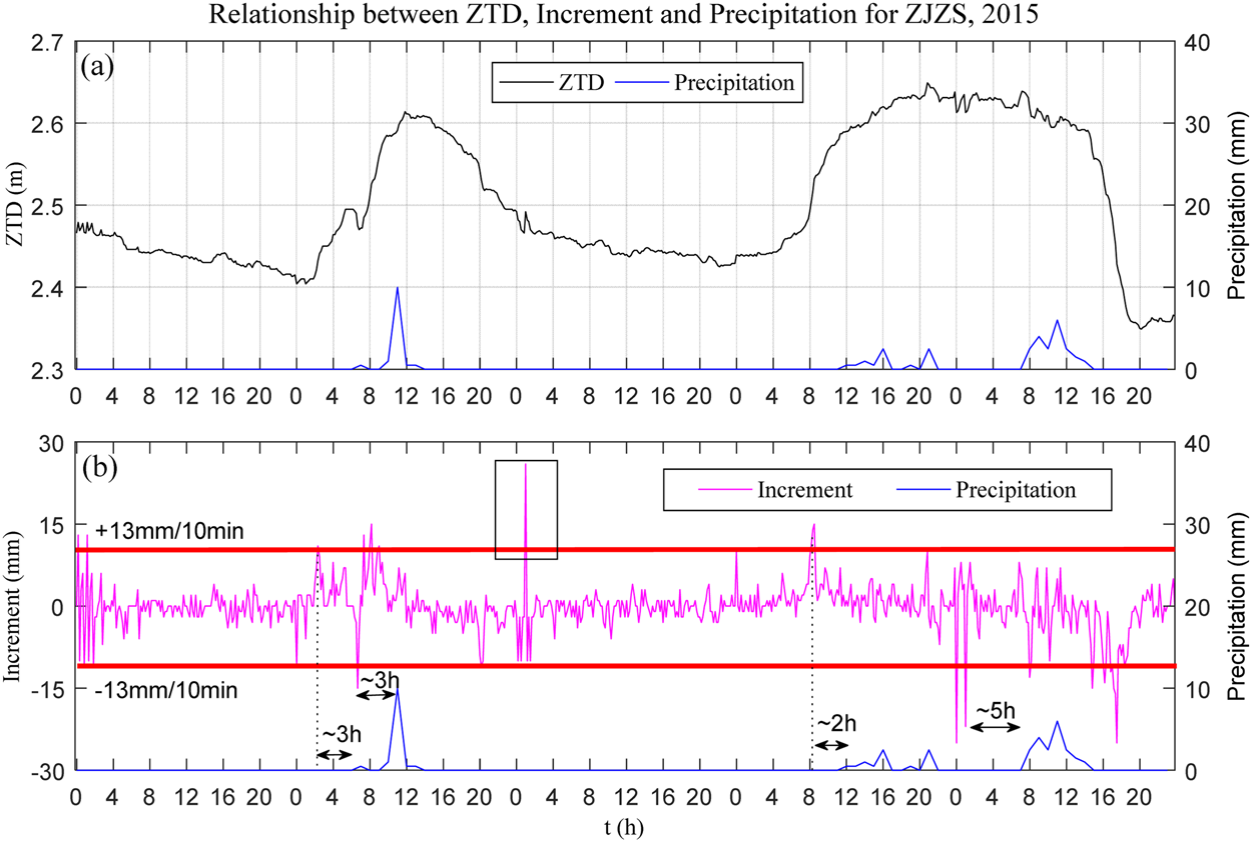
Comparison of ZTD, ZTD increment, and hourly accumulated precipitation at station ZJZS: 6–11 April, 2015. [the figure is plotted by MATLAB 2016a (https://cn.mathworks.com/products/matlab.html)].

**Figure 10:**
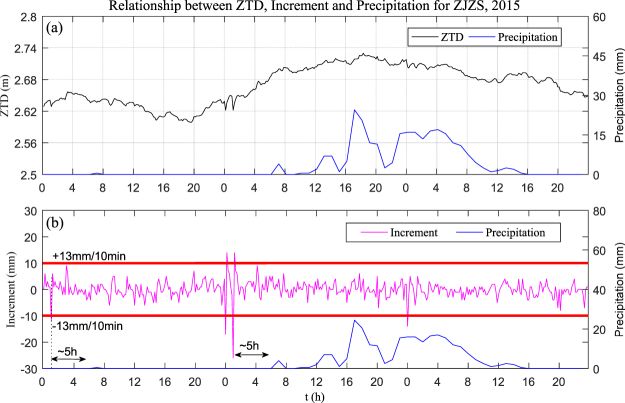
Comparison of ZTD, ZTD increment, and hourly accumulated precipitation at station ZJZS: 9–11 June, 2015. [the figure is plotted by MATLAB 2016a (https://cn.mathworks.com/products/matlab.html)].

Figures 9 and 10 show the relationship between ZTD, ZTD increment, and precipitation at station ZJZS for the two chosen time periods, respectively. Analysing the top plot in each of Figs 9 and 10, a ZTD time series for two periods is seen, where it should be noted that a continuous growth in ZTD before precipitation occurred, and precipitation happened when the ZTD reached its peak value. After precipitation, the ZTD value returned to its stable value, which suggested an atmospheric advection process. It also can be found, from the bottom plot of Figs 9 and 10, that the ZTD increment exceeded ±10 mm/10 min about 2 to 6 hours earlier than the rainstorm, but not all large ZTD increments were accompanied by precipitation (as shown in the black rectangle in Fig. 9(b)). Here, ZTD increment (mm/10 min) refers to the difference between the ZTD value of the current, and last epochs, which can reflect the variation in ZTD. It can also be seen that the ZTD peak arrived before the onset of precipitation. One explanation is that there was some time latency before precipitation struck the ground below; another possible explanation was that the satellite signal is more sensitive to water vaporous than to liquid water, and the decreasing value of ZTD indicated that vapour was being converted to liquid water or icy water. Therefore, such time latency between the variation of ZTD and precipitation just provides the possibility for precipitation forecasting.

According to the ten-minute ZTD increment, we judged whether, or not, precipitation occurred: the statistical results show that more than 86% of precipitation events happened when the ZTD increment exceed ± 10 mm/10 min within 2 to 6 hours before precipitation with a false alarm rate of 36.2%. From Figs 9 and 10 it can be observed that, when the ZTD value increased and reached its peak, precipitation generally occurred. Therefore, the ZTD slope as it increased could be considered as indicator of precipitation. Although the ZTD trend is not always smoothly increasing or decreasing as some studies mentioned previously with slightly fluctuation, a similar conclusion is derived: ZTD increases before precipitation, upon onset of rainfall, and then decreased after precipitation.

**Figure 11:**
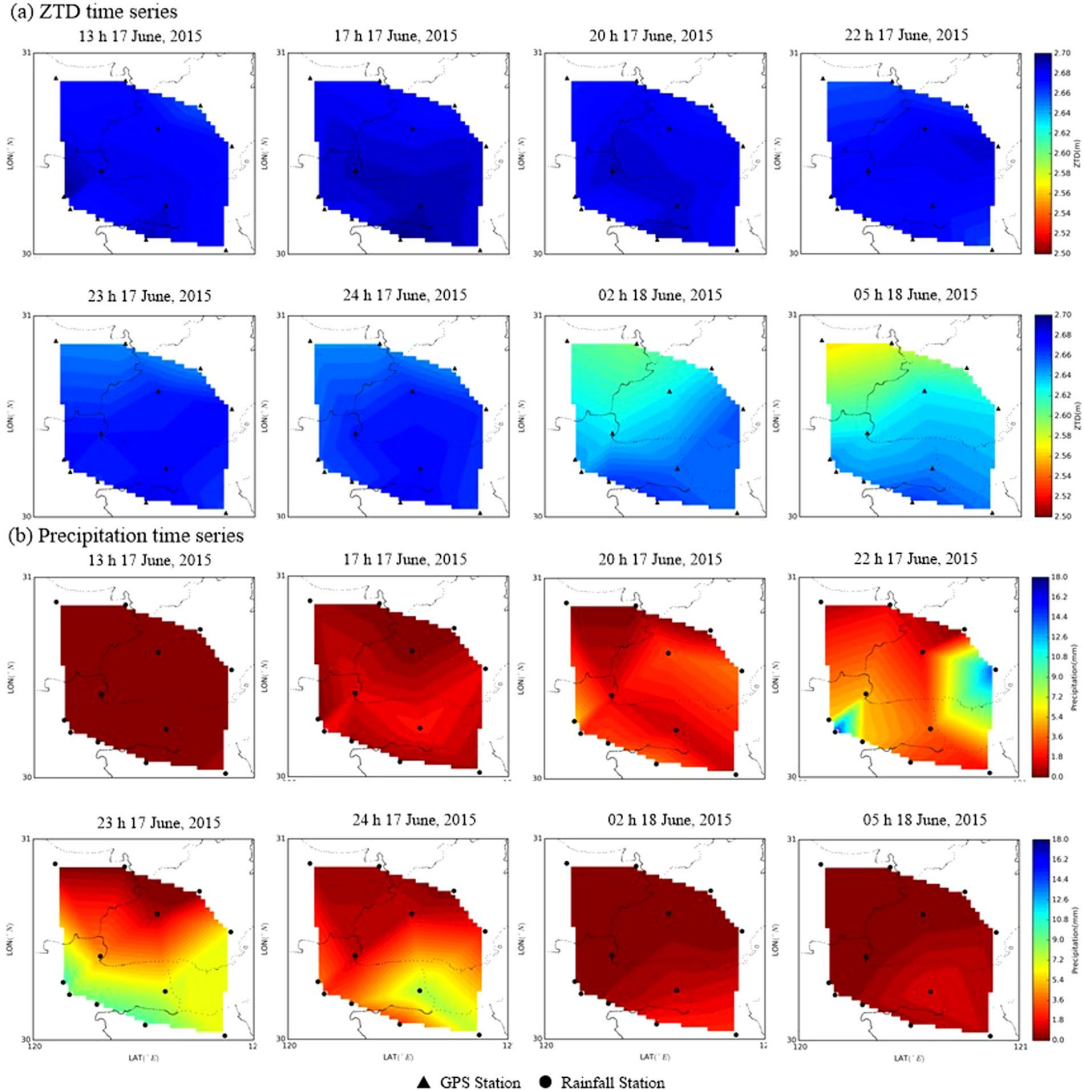
2-d interpolated maps of the temporal variation in ZTD for Zhejiang Province (top) compared with precipitation maps accumulated (bottom), 16 to 17 June, 2015. [the figure is plotted by MATLAB 2016a (https://cn.mathworks.com/products/matlab.html)].

In addition, for the cold front over the area of Zhejiang Province (as shown by magenta rectangle in Fig. 1) from 16 to 17 June, 2015, Fig. 11 shows the 2-d time series of ZTD and precipitation. It can be seen from Fig. 11 that a similar relationship between ZTD and precipitation existed with a strong ZTD increase with a maximum ZTD of up to 2.70 m before precipitation (13 h to 17 h, 17 June), the precipitation event itself with its maximum total precipitation of about 18 mm (20 to 22 h, 17 June), the decrease in ZTD upon the end of precipitation (2 h to 5 h, 18 June). It also can be seen from Fig. 10 that with the arrival of this cold front, the ZTD value was less than that before precipitation, reflecting a seasonal effect on the GPS delay^29^.

## A method for precipitation forecasting based on ZTD time series data

### Feasibility analysis of precipitation forecasting based on ZTD time series data

As mentioned above, the ZTD slope can be used for precipitation forecasting and the precipitation often happened after ZTD slope growth (usually within 2 to 6 hours thereafter). To analyse the feasibility of the proposed method, one full year of hourly ZTD at station ZJZS were analysed by a least-squares fitting method to obtain a broken line tendency of increases or decreases. A statistical experiment was performed which aimed to ascertain the number of well-forecasted precipitation events in the two to six hours before the ZTD slope exceeded a certain threshold and the total ZTD change as it increased (a few hours to more than ten hours, see Table 1). It can be seen from Table 1 that, with the increased threshold range of ZTD slope, ZTD slope became increasingly correlated with the number of well-forecast precipitation events. The percentage of well-forecast precipitation increased from 1.73% to 24.45%, evincing the positive correlation between ZTD slope and precipitation. Therefore, it is reasonable to consider ZTD slope as an indicator for precipitation forecasting.

**Table 1:** Relationship between ZTD slope and the number of rainfall events, unit: mm/h.

Threshold (mm)	[0 2]	[2 4]	[4 6]	[6 8]	[8 10]	>10
Precipitation times	16	35	32	26	29	56
ZTD slope	925	930	542	281	200	229
Percentage (%)	1.73	3.76	5.90	9.25	14.5	24.45

### Determination of threshold for ZTD slope

**Figure 12:**
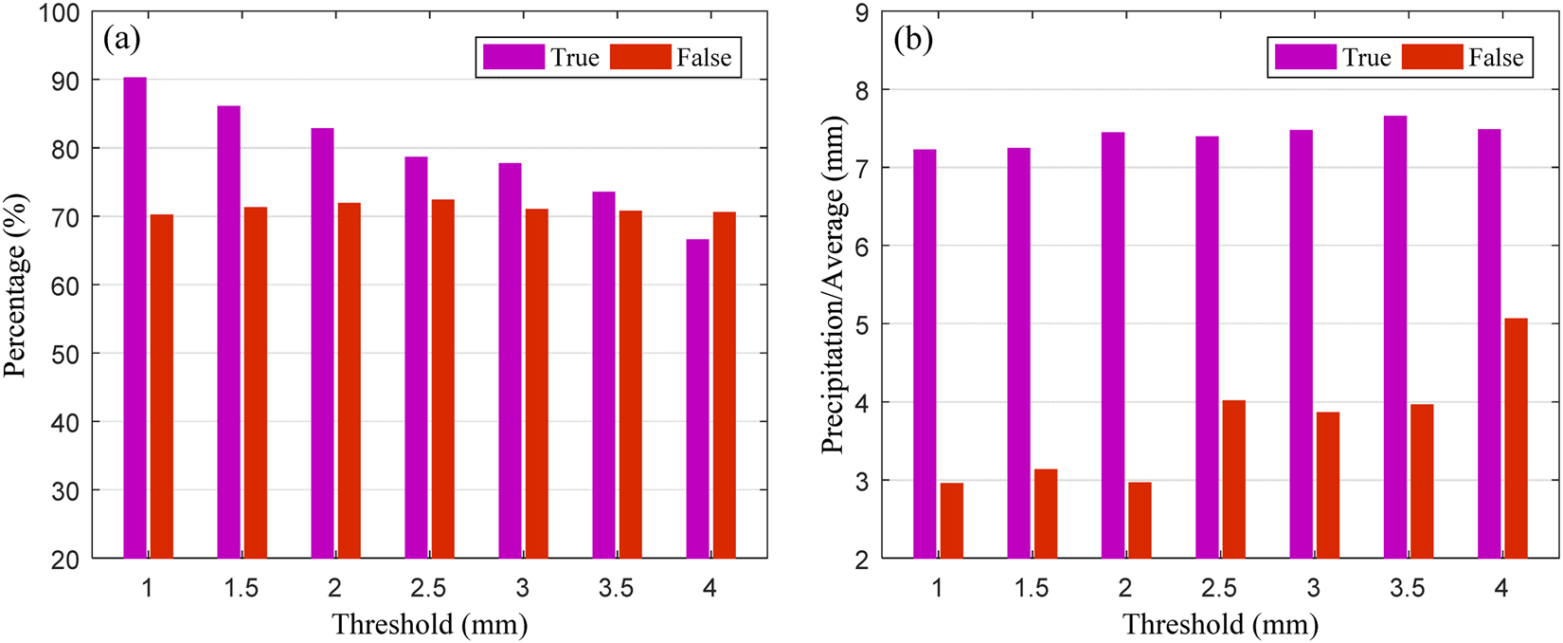
Relationship between ZTD slope and the percentage of forecast times, false alarms (left-hand plot), and mean accumulated rainfall in the well-, and badly-forecast precipitation events (right-hand plot). [the figure is plotted by MATLAB 2016a (https://cn.mathworks.com/products/matlab.html)].

The higher the threshold of ZTD slope, the more percentage of precipitation events can be forecasted (one point should, however, be noted: the actual number of well-forecast precipitation events is decreasing, because the total number of forecast precipitation events is reduced with increasing ZTD slope). Therefore, how to determine a reasonable threshold must be explored. In our study, the threshold of ZTD slope was analysed using a full year of hourly ZTD and hourly precipitation data. Figure 12 shows the change in percentage of well-forecast precipitation events, false alarms, average precipitation in those well-forecast precipitation events, and average precipitation in those missed precipitation events with different ZTD slopes.

Figure 12(a) shows that the percentage of well-forecast precipitation events decreased with the threshold of ZTD slope. The well-forecasted rate of precipitation events decreased from 90% to 70% while the false alarm rate being almost the same with increasing ZTD slope. From Fig. 12(b) it was found that the average value of well-forecast precipitation events was near-constant, while that of missed events tended to increase. When the ZTD slope was selected to be less than 2 mm/h, the average value of missed precipitation events was about 3 mm, and this increased to about 4 mm when the threshold level of the ZTD slope was 2.5 mm/h. Based on the above analysis, the threshold of ZTD slope was selected as 2 mm/h on the basis of a higher number of well-forecast precipitation events as well as the lower average value of the missed precipitation events.

### Validation of the precipitation forecasting method

To validate the reliability and stability of the proposed precipitation forecasting method, the selected threshold of ZTD slope mentioned above for station ZJZS was used using a full year of hourly PWV and precipitation event data. Numerical results showed that 196 out of 225 precipitation events were forecast based on the proposed method (a success rate of 87.11%). To validate the reliability of the proposed method, the hourly ZTD time series of the other nine stations from the CORS network in Zhejiang Province, and hourly accumulated precipitation data, were selected for the period 1 September, 2014 to 31 August, 2015. Table 2 shows the true rate and false alarm rate of the precipitation forecasting method. It can be seen that the average true rate of the forecasting method was between 80% and 90% while the false rate was between 60% and 70%. For the calculation involving data from ten stations, the average true rate was 85.18% which is far higher than that in a previous study based on the PWV values with its value of about 75%, while the average false rate was 66.00% which is almost the same as that of a previous study^29^. Therefore, the proposed precipitation forecasting method was deemed both effective and feasible. In addition, the false rates for ZJZS, ZJPH, and LJSL were relatively high, which is because those three stations are near the ocean and the variation in water vapour content thereat is greater. The large variation in water vapour content was embodied by the large change in ZTD, which leads to a relatively high false alarm rate.

**Table 2:** Statistical results: forecast rain times based on the proposed method for ten stations.

Station	True rate (%)	False rate (%)	Station	True rate (%)	False rate (%)
ZJZS	87.11	68.84	ZJWL	86.62	63.13
ZJJD	85.12	62.27	ZJXC	82.94	67.75
ZJCN	87.73	62.50	ZJXJ	88.89	66.43
ZJKH	83.57	62.74	ZJYH	83.51	63.72
ZJPH	83.48	70.78	LJSL	82.79	71.84

## Conclusion and Discussion

We evaluated the accuracy of GPS-derived ZTD value based on the independently developed PPP software using post-processed, and real-time, orbit and clock products provided by IGS and proposed a method for precipitation forecasting based on the ZTD slope during the time when it was continuously increasing. The post-processed GPS-derived ZTD was compared with the result derived from GAMIT software which based on the double-differenced model, Bernese PPP software which was based on the un-differenced model as well as the collocated independent observations of VLBI. The statistical result shows that the coefficients of post-processed GPS-derived ZTD were both greater than 0.98 and the RMS errors were less than 8 mm. For the period 25 June to 19 July, 2015, the RMS error in orbit accuracy was 4.79 cm and that of the clock accuracy was 0.38 ns for the real-time IGS products. The GPS-derived ZTD using IGS final, and real-time, orbit and clock products has been compared with half-year GPS observations at stations ZJJD and ZJZS for the period 1 February to 31 July, 2015. The corresponding real-time GPS-derived ZTD time series had a correlation coefficient greater than 0.99 and an RMS error of less than 10 mm with respect to the post-processed GPS-derived ZTD, which proved that the desired level of post-processed data accuracy can be reached under real-time processing conditions.

By comparing the relationship between the ZTD and PWV time series, we found that the continuous increase in ZTD was, in general, accompanied by the onset of rainfalls events, but not always. The reason for this may have been that the high ZTD level is only one of the prerequisites for the onset of precipitation, which is also triggered by some external dynamic factors. A method of precipitation forecasting was proposed based on the ZTD slope as it increased: this entailed analysing a full year of hourly PWV and hourly accumulated precipitation data from station ZJZS. Experimental testing of the method was undertaken using data from ten stations in the CORS network of Zhejiang Province for the purposes of validation. Numerical results showed that the proposed method could predict about 85% of the precipitation events in the year: this performance was superior to that achieved elsewhere by other studies, while the false alarm rate was about 66% which was equivalent to the level obtained by others. Currently, there was about 15% of precipitation events cannot be predicted.

Although the real-time GPS-derived ZTD estimation could not, so far, be considered as a stand-alone system for precipitation forecasting, as some other external dynamic factors were not considered; however, the real-time GPS-derived ZTD values could be used by way of their assimilation into existing forecasting systems where they would be expected to enhance the forecasting capabilities of the existing system. In addition, the present method only used one single station for precipitation forecasting, if more stations were included and three-dimensional analysis (two-dimensional in space and evolving in time) were to have been undertaken, the real-time GPS-derived ZTD forecasting method may be able to become a stand-alone system used for nowcasting.
